# Prevalence and correlates of obesity among Lusaka residents, Zambia: a population-based survey

**DOI:** 10.1186/1755-7682-5-14

**Published:** 2012-05-02

**Authors:** Emmanuel Rudatsikira, Adamson S Muula, David Mulenga, Seter Siziya

**Affiliations:** 1 School of Community Health and Environmental Health, College of Health Sciences, Old Dominion University, Norfolk, USA; 2 Department of Community Health, College of Medicine, University of Malawi, Blantyre, Malawi; 3 Public Health Unit, Department of Clinical Sciences, School of Medicine, The Copperbelt University, Ndola, Zambia

**Keywords:** Obesity, Smoking, Lusaka, Zambia

## Abstract

**Background:**

Non-communicable lifestyle diseases are a growing public health concern globally. Obesity is a risk factor for premature mortality from cardiovascular diseases and diabetes as well as all-cause mortality. The objective of the study was to estimate the prevalence and associated factors for obesity among Zambian adults in Lusaka district.

**Methods:**

A community-based study was done among adults in Zambia. Descriptive and co-relational analyses were conducted to estimate the prevalence of being obese as well as identify associated factors.

**Results:**

A total of 1,928 individuals participated in the survey, of which 33.0% were males. About half of the participants were aged 25–34 years (53.2%), and about two-thirds had attended at least secondary level of education (63.9%). Overall, 14.2% of the participants (5.1% of males, and 18.6% of females) were obese. Significant factors associated with obesity were sex, age, education, cigarette smoking and blood pressure. Male participants were 55% (AOR = 0.45; 95% CI [0.29, 0.69]) less likely to be obese compared to female participants. Compared to participants who were of age 45 years or older, participants of age 25–34 years were 61% (AOR = 0.39 (95% CI [0.23, 0.67]) less likely to be obese. Compared to participants who attained college or university level of education, participants who had no formal education were 63% (AOR = 0.37; 95% CI [0.15, 0.91]) less likely to be obese; and participants who had attained secondary level of education were 2.22 (95% CI [1.21, 4.07]) times more likely to be obese. Participants who smoked cigarettes were 67% (AOR = 0.33; 95% CI [0.12, 0.95]) less likely to be obese compared to participants who did not smoke cigarettes. Compared to participants who had severe hypertension, participants who had moderate hypertension were 3.46 (95% CI [1.34, 8.95]) times more likely to be obese.

**Conclusions:**

The findings from this study indicate that Zambian women are more at risk of being obese. Prevention and control measures are needed to address high prevalence and gender inequalities in risks for non-communicable diseases in Zambia. Such measures should include policies that support gender specific approaches for the promotion of health behavior changes.

## Background

Obesity is a risk factor for the metabolic syndrome and premature mortality from cardiovascular diseases (CVD) and all-cause mortality [1-3]. By 2020 it is estimated that CVD will become the leading cause of the global health burden, accounting for 73 percent of total global mortality and 56 percent of total morbidity [4]. However, the risk of death has been inconsistent [5,6]. Based on previous understanding on the epidemiologic transition, non-communicable diseases (NCD) have largely been viewed as diseases of affluent societies or high-income countries. However, current evidence suggests that the prevalence of cardiovascular diseases, diabetes and risk factors such as obesity have been just as high in low-income nations as is in high-income nations. Even in high-income nations, there is disparate in both ill-health and mortality in that risk factors such as obesity, cardiovascular and metabolic syndrome, and related mortality are more common among people within the lower socio-economic strata.

The World Health Assembly and the United Nations General Assembly have identified non-communicable diseases such as cardiovascular diseases as critical public health problems. A number of low-income countries, which had for decades only prioritized infections, vaccine-preventable childhood illnesses and the traditional diseases of poverty such as under-nutrition, diarrhoea and schistosomiasis, are now addressing the epidemic of non-communicable diseases. For example, Malawi has recently included non-communicable diseases (such as stroke, and diabetes) in its Essential Health Programme (Ministry of Health [Malawi], 2011). This upgrade means that primary care and prevention should be available to all people with these conditions in Malawi.

Available data suggest that Zambia, like many other low-income countries may be experiencing an upsurge of non-communicable diseases. Goma et al. [7] have reported a prevalence of hypertension of 34.8% (38.0% of males and 33.3% of females) among Lusaka residents. Nsakashalo-Senkwe et al. [8] estimated a combined prevalence for impaired glucose level or diabetes of 4.0%. In order to inform public health policy and practice, there is need to quantify the prevalence and associated factors for obesity in Zambia.

## Methods

### Study setting

A national survey was planned. However, we were too ambitious to conduct a survey of that magnitude. The limiting factor was funding. In 2007, the survey was conducted in Lusaka district. A year later, it was repeated in Kaoma and Kasama rural districts. Up to date, only three of the eight originally sampled districts have been surveyed. The survey from which this report was derived was conducted in Lusaka, Zambia. Lusaka is the capital of Zambia with a population of 2,198,996 people (CSO, 2010 Census of population and housing. Preliminary report).

### Study design

The data from which this study is generated were collected through a community-based cross sectional survey, using a modified WHO global NCD surveillance initiative NCD-STEP 3 [9].

### Sample size and sampling

The required sample size for the national survey was computed using the Statcal programme in Epi Info version 6.04. We obtained a sample size of 7660 upon considering a 95% confidence level, 5% margin of error, prevalence of 50%, 8 provinces, a design effect of 2, and an 80% response rate. A total of 1915 participants were to be sampled from Lusaka district. The study was powered to produce estimates of NCDs at district level.

A district in Zambia is divided into constituencies, then into wards, followed by Census Supervisory Areas (CSAs), and finally into Standard Enumeration Areas (SEAs). A listing of constituencies, wards and SEAs based on the 2000 census was obtained from the Central Statistical Office (CSO). Lusaka district had 7 constituencies, out of which 5 were randomly selected. From each selected constituency, one ward was randomly selected. The number of Standard Enumeration Areas (SEAs) selected in each ward was proportional to its population size. SEAs were selected using a systematic random sampling method. Finally, households were systematically sampled in each selected SEA and all persons of ages 25 or more years were requested to participate in the survey.

### Ethical considerations

The study protocol was approved by the University of Zambia (UNZA) Research Ethics Committee (REC). Permission to conduct the study was given by the Ministry of Health [Zambia]. Interviews were conducted in the homes of the participants, and waist measurement was taken in a private area.

### Data collection

Data collection was done in 2007. Research assistants (2 nurses, 2 laboratory technologists, 1 social science undergraduate student, 1 pharmacy diploma student, 1 surveyor from the Central Statistical Office, and 1 high school graduate) were trained by a physician who is a physiologist to take anthropometric measurements and blood pressure readings. In addition, the research assistants were also trained in administering the questionnaire. Training of research assistants took five days. On the fourth day, a pilot study was conducted and investigators were satisfied that the research assistants were to collect good quality data. The fifth day was spent on making changes mainly on how the questions were to be asked in local languages, and duplicating and collating the questionnaires. Data collection was conducted in the homes of the participants.

### Height

Height was measured using the Seca 214 portable stadiometer (Seca gmbh & Co. kg Humburg, German). While taking the measurement, participants were requested to remove foot wear or head gear, have feet together, heels against the back board, knees straight, and look straight ahead. Height was recorded in centimetres.

### Weight

Weight was measured using the Heine 737 portable professional adult scale (Seca gmbh & Co. kg Humburg, German). Participants were asked to stand still, face forward, and place arms on the sides of the body. Weight was recorded in kilograms.

### Blood pressure

Blood pressure was measured three times using the OMRON digital automatic BP monitor M4-1 (OMRON Healthcare Europe BV, The Netherlands). Participants rested for three minutes in between taking the measurements. An average of the three readings was considered as the blood pressure for the participant.

### Definitions

Body mass Index (BMI) was categorized as <18.5 kg/m^2^ (lean), 18.5–24.9 kg/m^2^ (normal), 25.0–29.9 kg/m^2^ (overweight), and 30+ kg/m^2^ (obese). A participant with blood pressure of more than 140/90 mmHg was classified as being hypertensive. Age was collected as a continuous variable but was categorized into the age groups 25–34, 35–44, and 45+ years during the analysis as recommendation by the World Health Organization on how to analyze NCDs data (WHO, 2005). Systolic blood pressure was grouped into four levels: <140 (normal), 140–169 (mild hypertension), 170–179 (moderate hypertension), and 180+ (severe hypertension); similarly diastolic blood pressure was grouped into four levels: <90 (normal), 90–99 (mild hypertension), 100–109 (moderate hypertension), and 110+ (severe hypertension). A participant with a severe systolic blood pressure reading or severe diastolic blood pressure was classified as having severe hypertension. Moderate hypertension on either systolic or diastolic blood pressure categorized a participant as having moderate hypertension. Mild hypertension on either systolic or diastolic blood pressure categorized a participant as having mild hypertension. A participant was classified as having norm tension when both systolic and diastolic blood pressure was normal.

### Data analysis

We conducted both bivariate and multivariate analyses. A multivariate logistic regression was used to determine independent predictors for obese for each gender. Unadjusted odds ratios (OR) and their 95% confidence interval (CI), and adjusted odds ratios (AOR) and their 95% CI are presented.

## Results

A total of 1928 individuals participated in the survey, of which 33.0% were males. About half of the participants were aged 25–34 years (53.2%), and about two-thirds had attended at least secondary level of education (63.9%). Overall, 14.2% of the participants (5.1% of males, and 18.6% of females) were obese. These results are shown in Table 1.

**Table 1:** Distributions of socio-demographic characteristics and obesity between sexes in the Lusaka Survey 2007

	Total	Male	Female
Factor	n (%)	n (%)	n (%)
**Age group (years)**			
25–34	1015 (53.2)	337 (53.7)	675 (52.9)
35–44	413 (21.6)	135 (21.5)	277 (21.7)
45+	481 (25.2)	156 (24.8)	323 (25.3)
**Sex**			
Male	634 (33.0)	-	-
Female	1288 (67.0)	-	-
**Education**			
None	408 (21.5)	76 (12.2)	330 (26.0)
Primary	276 (14.5)	61 (9.8)	214 (16.9)
Secondary	679 (35.8)	242 (38.8)	435 (34.3)
College/university	534 (28.1)	244 (39.2)	290 (22.9)
**Obesity**			
Yes	269 (14.2)	32 (5.1)	236 (18.6)
No	1632 (85.8)	595 (94.9)	1032 (81.4)

Table 2 shows factors associated with obesity. Significant factors associated with obesity in bivariate analyses were sex, age, education, eating vegetables, vigorous-intensity activities, cigarette smoking and blood pressure. However, in a multivariate analysis, eating vegetables and vigorous-intensity activities were no longer significantly associated with obesity. Male participants were 55% (AOR = 0.45; 95% CI [0.29, 0.69]) less likely to be obese compared to female participants. Compared to participants who were of age 45 years or older, participants of age 25–34 years were 61% (AOR = 0.39 (95% CI [0.23, 0.67]) less likely to be obese. In terms of education, compared to participants who had attained college or university level of education, participants who had no formal education were 63% (AOR = 0.37; 95% CI [0.15, 0.91]) less likely to be obese; and participants who had attained secondary level of education were 2.22 (95% CI [1.21, 4.07]) times more likely to be obese. Participants who smoked cigarettes were 67% (AOR = 0.33; 95% CI [0.12, 0.95]) less likely to be obese compared to participants who did not smoke cigarettes. Compared to participants who had severe hypertension, participants who had moderate hypertension were 3.46 (95% CI [1.34, 8.95]) times more likely to be obese.

**Table 2:** Factors associated with obese among Lusaka residents, Zambia-2007

Factor	Odds Ratio (95% Confidence Interval)	
	Unadjusted	Adjusted
**Sex**		
Male	**0.48 (0.40, 0.59)**	**0.45 (0.29, 0.69)**
Female	1	1
**Age group (years)**		
25–34	**0.49 (0.41, 0.59)**	**0.39 (0.23, 0.67)**
35–44	1.12 (0.92, 1.37)	1.28 (0.76, 2.15)
45+	1	
**Education**		
None	0.90 (0.70, 1.15)	**0.37 (0.15, 0.91)**
Primary	1.21 (0.94, 1.57)	1.76 (0.82, 3.77)
Secondary	**0.79 (0.64, 0.98)**	**2.22 (1.21, 4.07)**
college/university	1	1
**Number of days ate vegetables in a typical week**		
0–4	**0.57 (0.36, 0.90)**	0.51 (0.16, 1.61)
5–7	1	1
**Number of days ate fruits in a typical week**		
0–4	1.04 (0.89, 1.22)	0.92 (0.62, 1.37)
5–7	1	1
**Number of hours spent on sitting or reclining on a typical day (Sedentary behaviour)**		
<1.5	0.86 (0.69, 1.06)	0.70 (0.39, 1.27)
1.5–3.4	1.14 (0.96, 1.36)	1.15 (0.70, 1.88)
3.5+	1	1
**Vigorous-intensity activity for 10 minutes continuously**		
Yes	**0.76 (0.61, 0.94)**	0.72 (0.41, 1.28)
No	1	1
**Consumed alcohol in past 30 days**		
Yes	0.92 (0.66, 1.30)	1.20 (0.78, 1.85)
No	1	1
**Smoked cigarettes in past 30 days**		
Yes	**0.37 (0.21, 0.65)**	**0.33 (0.12, 0.95)**
No	1	1
**Blood pressure**		
Normal	**0.26 (0.16, 0.41)**	0.73 (0.35, 1.52)
Mild	0.65 (0.40, 1.04)	1.67 (0.79, 3.52)
Moderate	1.27 (0.72, 2.24)	**3.46 (1.34, 8.95)**
Severe	1	1

## Discussion

In a study of community-based adults in Lusaka, Zambia, 14.2% (5.1% among male and 18.6% among female) participants were obese. These figures are higher than those reported in Malawi by Msyamboza et al. [10] who found that 2.0% of men and 7.3% of women were obese. The prevalence of obesity was 11.1% (7.5% among males, and 21.2% among females) in Douala, Cameroon [11]; and in four urban districts of Cameroon (Yaounde, Douala, Garoua and Bamenda) the prevalence of obesity were 6.5% in males and 19.5% in females [12] and accord our findings. Our results are also comparable to those found among urban residents in Tanzania and Namibia of 17.4% and 17.0%, respectively [13]. However, obesity rates in our study are lower than those reported in urban adult populations in South Africa of 9.2% among males and 42% among females [14], and lower than 30.6% reported in the United States between 1999 and 2002 among persons in the 25–59 years age group [5].

With regard to the factors that were assessed if they were associated with being obese, male gender, aged 25–34 years, no education and smoking were negatively associated with being obese. Meanwhile, secondary level of education and moderate hypertension were positively associated with obesity. Our finding that males were less likely to be obese has also been reported by Bernabe-Ortiz et al. [15] who observed that females were more likely to be moderately physically active, whereas males were more likely to report heavy physical activity. While most (69.4%) women aged 15–49 years in Lusaka province were engaged in sales and services occupational sectors, most male residents in the same age group were engaged in sales and services (39.8%) and skilled manual (29.8%) [16]. A similar finding was also reported in Malawi [10]. This may have biological reasons, but also lifestyle and cultural reasons. For instance, in a study reported by Bentley et al. [17], Malawian women perceived larger body shapes as healthy, interpreting fatness as a sign of good health and absence of disease. We are not aware of any similar study from Zambia. However, if Zambians also perceive being large as a marker of good health, this may impede their adoption of lifestyles that would enable them lose weight. Several theories of health behaviour change e.g. Health Belief Model, Trans-theoretical model, Social Cognitive theory [18-20], all suggest that change is more likely to happen if/when the concerned individual understands that there is a problem that needs changing.

We found that participants who were in the 25–34 years age group were less likely to be obese compared to older participants aged 45 years or older. A similar finding has been reported by Hedley et al. [21] in the United States. The 25–34 year age group as young adults may be more conscious of the body image such that they may take dietary or physical activity measures to control their weight. Further, as people grow old, their daily routines may change towards having less free time for exercise or other physical activities which could impact their weight. Lusaka is the commercial town of Zambia. The majority of people are not engaged in strenuous physical labour to earn their living. Without deliberate effort to exercise or engage in other physical activity, weight gain is likely for most of the residents especially if daily energy intake remains unchanged or increases.

In the current study, respondents without formal education were less likely to be obese. This finding contradicts that of Salonen et al. [22] who found that lower educational attainment was associated with higher body mass index. Education is one of the indicators of socioeconomic status [23,24]. Although, higher educational attainment is associated with increased knowledge that in turn enables an individual to make healthy choices [25], in the Zambian culture, obesity especially among males may be regarded as a symbol of wealth.

We found that smokers were less likely to be obese compared to non smokers. Similar findings have been reported elsewhere [26-28]. Smoking reduces appetite and stimulates the body’s metabolic rate [29]. People may use smoking to reduce weight. Data from especially adolescent girls suggest that smoking may be used to control weight gain among this age group.

Although we found that participants with moderate hypertension were more likely to be obese compared to participants with severe hypertension, evidence suggests that obesity contributes to hypertension such that controlling obesity may eliminate 48% of the hypertension in whites and 28% in blacks [30]. It is important to note that patients who present with both hypertension and obesity, also present with other unfavourable conditions for cardiovascular prognosis [31].

### Limitations of the study

The current study has several limitations. Firstly data were collected cross sectionally. We cannot therefore ascribe causation on the relations of the variables found associated with the outcome measure (obesity). We did not obtain information to enable us to calculate weights. However, we believe that our sample was representative of the Lusaka population given the multistage sampling method that we used. Information on marital status was not available, and our findings may have been biased if marital status was a confounding factor. However, a study conducted in Helsinki, Finland did not find a significant association between marital status and obesity [22]. Considering the heterogeneity in individual body fatness and the associated risk for co-morbidities at a given BMI, the overall individual risk of morbidity or mortality may differ even at the same BMI [32]. Further, previous literature suggests that the heterogeneity in BMI is related not only to age and sex, but also ethnicity and differences in body composition and body-fat distribution. An assessment of total body fat could possibly add more information to the estimation of adverse health risk than BMI alone. In addition, BMI might not correlate well with body fat resulting from age, sex, and ethnic factors. As an assessment tool for body fatness, BMI has only a sensitivity of 50%, although specificity is much higher at 90% in identifying excess adiposity [32]. In addition, the socio-demographic characteristics were self –reported. To the extent that study participants misreported, either intentionally or inadvertently, the findings may be biased.

## Conclusions

The findings from this study indicate that Zambian women are more at risk of being obese. Prevention and control measures are needed to address high prevalence and gender inequalities in risks for non-communicable diseases in Zambia. Such measures should include policies that support gender specific approaches for the promotion of health behavior changes.

## Competing interests

The authors declare that they have no competing interest.

## Authors’ contributions

ER participated in the interpretation of the results and participated in drafting the manuscript. ASM participated in the interpretation of the results and led the drafting of the manuscript. DM participated in the interpretation of results and drafting of manuscript. SS participated in the design of the study, supervised data collection, performed the statistical analysis, participated in the interpretation of the results, and participated in drafting the manuscript. All authors read and approved the final manuscript.
